# Supplementary material for: Pan-Cancer Analysis and Experimental Validation Identify ACOT7 as a Novel Oncogene and Potential Therapeutic Target in Lung Adenocarcinoma
Source: Cancers (Basel). 2022 Sep 18;14(18):4522. doi: 10.3390/cancers14184522 (PMC9497106; doi:10.3390/cancers14184522)
Supplement: Supplementary file 1 [file cancers-14-04522-s001.zip › cancers-1834435-supplementary figures.pdf]

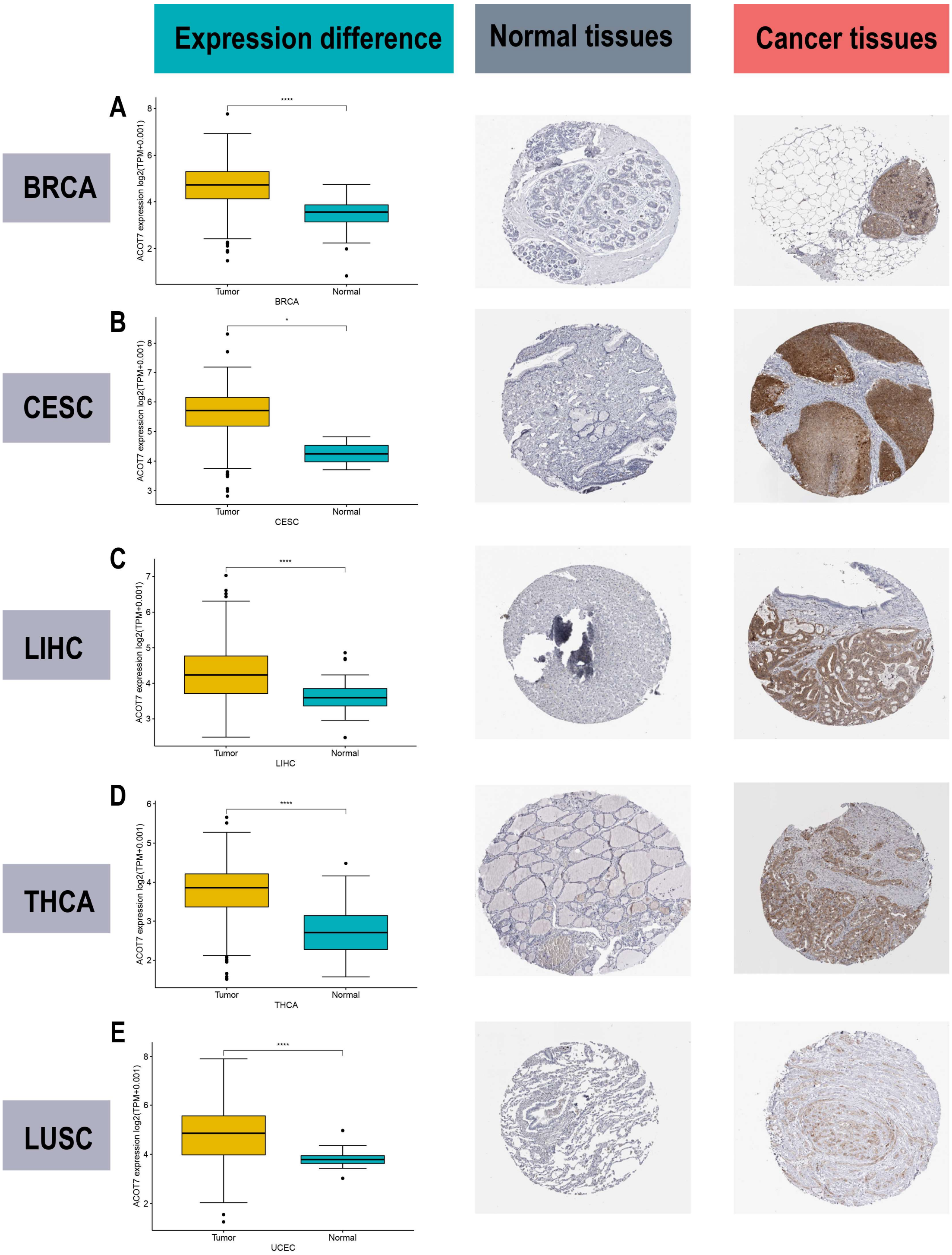

Figure S1. Comparison of ACOT7 expression between normal and tumor tissues with corresponding immunohistochemistry images. (A) Breast. (B) Cervix. (C) Liver. (D) Thyroid. (E) Lung. Notes: * $p < 0.05$, **** $p<0.0001$.

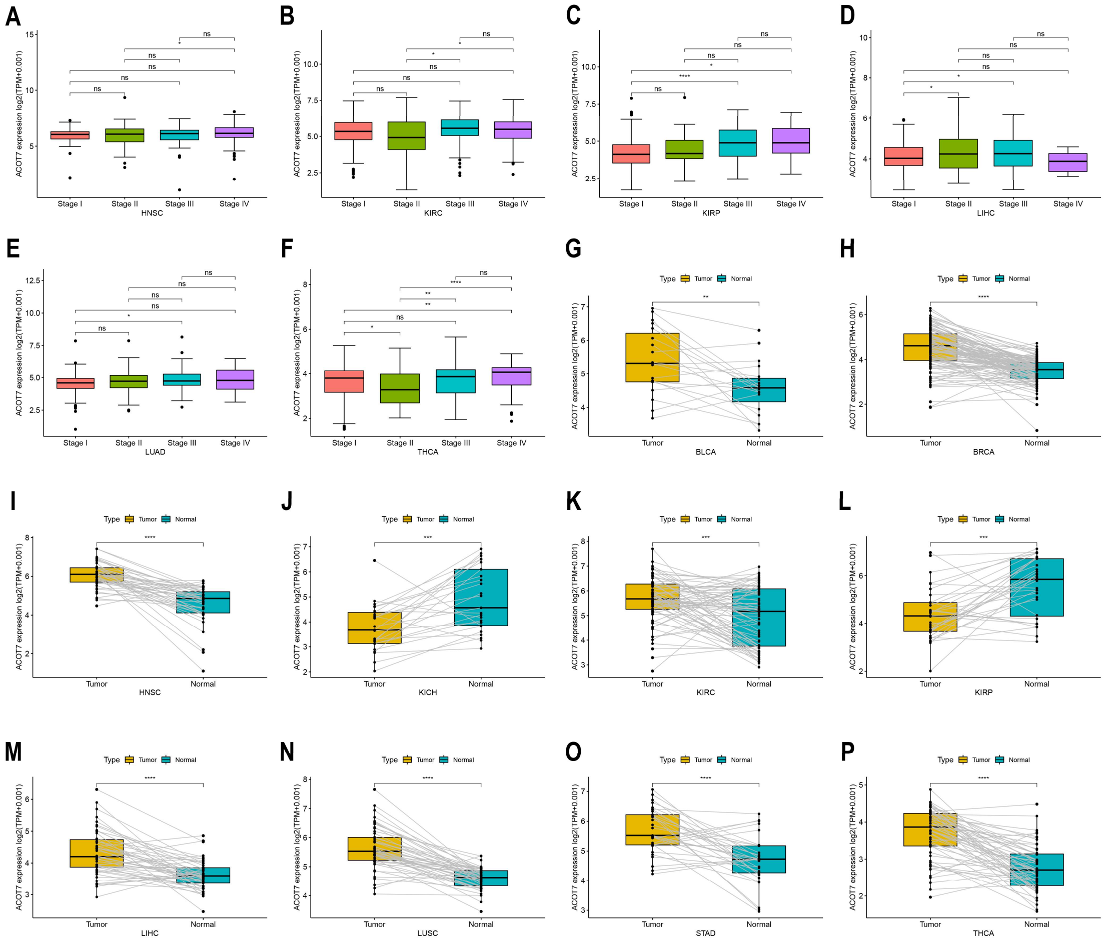

Figure S2. ACOT7 mRNA expression in pan-cancer. Association between ACOT7 expression and TNM stage in (A) HNSC, (B) KIRC, (C) KIRP, (D) LIHC, (E) LUAD, and (F) THCA; difference in ACOT7 expression in paired normal and tumor tissues in (G) BLCA, (H) BRCA, (I) HNSC, (J) KICH, (K) KIRC, (L) KIRP, (M) LIHC, (N) LUSC, (O) STAD, and (P) THCA. Notes: * $p < 0.05$, ** $p < 0.01$, *** $p < 0.001$, **** $p<0.0001$.

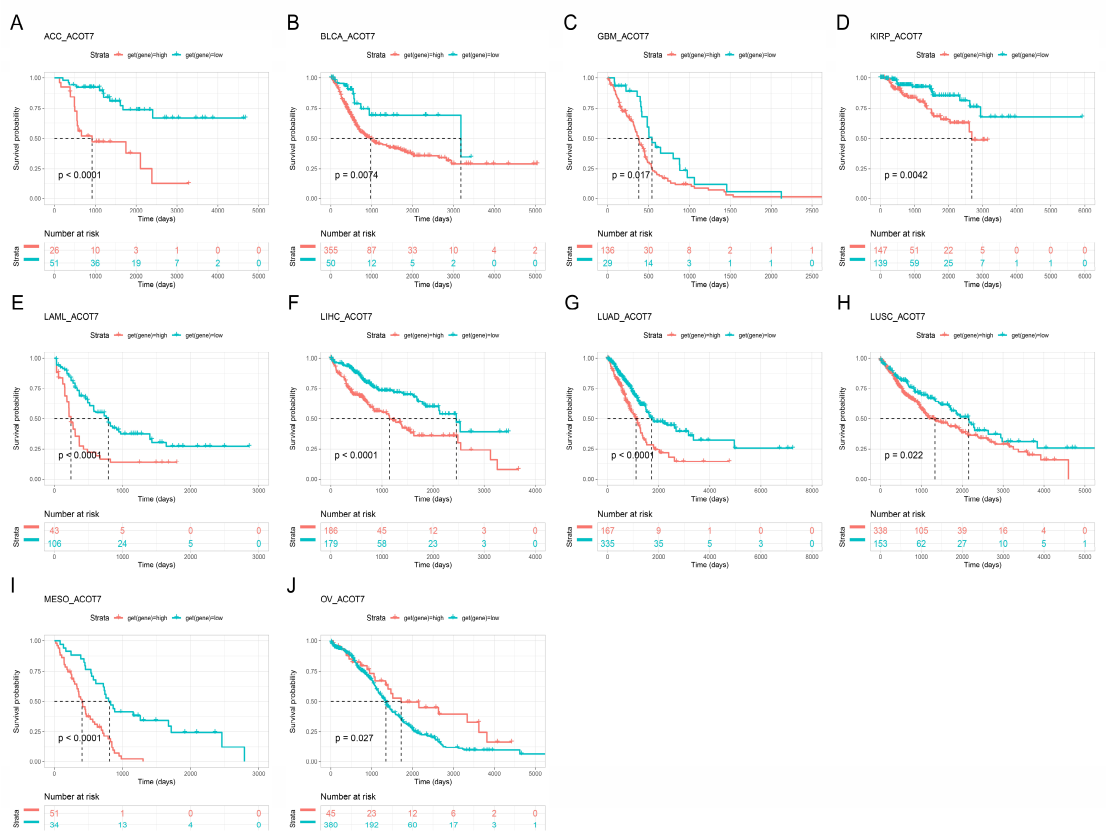

Figure S3. Kaplan–Meier curves of patients with different ACOT7 expression in 10 cancers including (A) ACC, (B) BLCA, (C) GBM, (D) KIRP, (E) LAML, (F) LIHC, (G) LUAD, (H) LUSC, (I)MESO, and (J) OV.
