# Supplementary material for: Pan-Cancer Analysis and Experimental Validation Identify ACOT7 as a Novel Oncogene and Potential Therapeutic Target in Lung Adenocarcinoma
Source: Cancers (Basel). 2022 Sep 18;14(18):4522. doi: 10.3390/cancers14184522 (PMC9497106; doi:10.3390/cancers14184522)
Supplement: Supplementary file 1 [file cancers-14-04522-s001.zip › Table S1.pdf]

Supplementary Table S1. Characteristic information of 33 types of cancer included in this study.

Abbreviation	Full name	Number of samples	
		Tumor	Normal
ACC	Adrenocortical carcinoma	77	0
BLCA	Bladder urothelial carcinoma	407	19
BRCA	Breast invasive carcinoma	1098	113
CESC	Cervical squamous cell carcinoma and endocervical adenocarcinoma	306	3
CHOL	Cholangiocarcinoma	36	9
COAD	Colon adenocarcinoma	288	41
DLBC	Lymphoid neoplasm diffuse large B-cell lymphoma	47	0
ESCA	Esophageal carcinoma	182	13
GBM	Glioblastoma multiforme	165	0
HNSC	Head and neck squamous cell carcinoma	520	44
KICH	Kidney chromophobe	66	25
KIRC	Kidney renal clear cell carcinoma	531	72
KIRP	Kidney renal papillary cell carcinoma	289	32
LAML	Acute myeloid leukemia	173	0
LGG	Brain lower-grade glioma	522	0
LIHC	Liver hepatocellular carcinoma	371	50
LUAD	Lung adenocarcinoma	515	59
LUSC	Lung squamous cell carcinoma	498	50
MESO	Mesothelioma	87	0
OV	Ovarian serous cystadenocarcinoma	427	0
PAAD	Pancreatic adenocarcinoma	179	4
PCPG	Pheochromocytoma and paraganglioma	182	3
PRAD	Prostate adenocarcinoma	496	52
READ	Rectum adenocarcinoma	92	10
SARC	Sarcoma	262	2
SKCM	Skin cutaneous melanoma	496	1
STAD	Stomach adenocarcinoma	414	36
TGCT	Testicular germ cell tumors	137	0
THCA	Thyroid carcinoma	512	59
THYM	Thymoma	119	2
UCEC	Uterine corpus endometrial carcinoma	181	13
UCS	Uterine carcinosarcoma	57	0
UVM	Uveal melanoma	79	0
Total		9784	712
