# Supplementary material for: Pan-Cancer Analysis and Experimental Validation Identify ACOT7 as a Novel Oncogene and Potential Therapeutic Target in Lung Adenocarcinoma
Source: Cancers (Basel). 2022 Sep 18;14(18):4522. doi: 10.3390/cancers14184522 (PMC9497106; doi:10.3390/cancers14184522)
Supplement: Supplementary file 1 [file cancers-14-04522-s001.zip › Table S2.pdf]

Table S2. Relationship between ACOT7 expression and immune cell infiltration in eight cancers.

Symbol	BLCA		BRCA		HNSC		KIRC		LUAD		STAD		THCA		THYM	
	p	r	p	r	p	r	p	r	p	r	p	r	p	r	p	r
CD4_naive	***	-0.19		0.03	**	0.13	***	-0.15		-0.05		-0.10	***	-0.31	*	0.23
CD8_naive	***	-0.22		-0.04		-0.01	***	-0.40		-0.05	*	-0.10	***	-0.25	***	0.61
Tc	***	0.30		0.03		-0.04	***	0.18	**	0.13	***	0.19	***	0.33		-0.03
Tex	***	0.38	***	0.19	*	0.10	***	0.30	***	0.18	***	0.31	***	0.20		0.12
Tr1	*	0.11	**	0.10	***	-0.18	***	0.19		-0.02		-0.09	***	0.33		0.01
nTreg	**	0.14	**	0.08	***	0.18	**	0.13	***	0.28	***	0.28	***	0.42	***	-0.30
iTreg	***	0.27	***	0.16		-0.04	***	0.17	***	0.20	*	0.10	***	0.44		-0.14
Th1	***	0.37	***	0.14	**	0.12	***	0.26	***	0.18	***	0.26	***	0.30		-0.06
Th2		0.08		0.02	**	-0.13	***	0.17		-0.06	***	-0.20		0.05	**	-0.28
Th17	***	-0.19	***	-0.27		0.03	***	-0.15	***	-0.27	***	-0.24	***	-0.16		0.15
Tfh	*	-0.11	*	-0.06	***	-0.23	***	0.27	*	-0.11	**	-0.13	***	0.33	***	0.48
Tcm		0.03	**	-0.09	**	-0.13	***	0.18		-0.02		-0.05	***	0.23	***	0.41
Tem		0.06	***	0.11	***	0.15		0.04		-0.01		0.03	***	0.16	***	-0.34
NKT		0.09	***	-0.21		0.02		0.08		0.00		-0.04	***	0.35	***	-0.62
MAIT		-0.08		-0.05		-0.03	***	-0.17	***	-0.16	***	-0.28	***	-0.32		0.05
DC	***	0.38	***	0.20	***	0.17		0.07	**	0.14	***	0.22	***	0.52	***	0.52
B_cell	*	-0.12	***	0.11	***	-0.22	***	-0.15	*	-0.11	*	-0.12	***	-0.16	***	0.34
Monocyte	***	0.18	***	0.10		0.08		0.04	***	0.17	*	0.11	***	0.24	***	-0.58
Macrophage	***	0.39	***	0.13		0.07	***	0.17	***	0.15		0.08	***	0.31	***	-0.49
NK	***	0.21		-0.02		-0.04	***	0.23		0.01		0.01	***	0.21	***	-0.42
Neutrophil		-0.09		0.00	***	0.22	***	-0.19		0.00	**	0.15	***	-0.44	***	-0.39
Tgd	***	-0.22	***	-0.13	***	-0.23	***	0.20	*	-0.09		0.07	***	-0.20		-0.02
CD4_T	***	-0.35	***	-0.13	***	-0.25		-0.06	***	-0.18	***	-0.35	*	0.11	***	0.32
CD8_T		-0.04		-0.02	***	-0.17	***	0.30		0.01		0.07	***	0.18	***	0.50
InfiltrationScore	***	0.32	***	0.14		-0.04	***	0.26	**	0.12		0.09	***	0.43	***	-0.32

Notes: $*P < 0.05$; $**P < 0.01$; $***P < 0.001$.
